# An Efficient LCM-Based Method for Tissue Specific Expression Analysis of Genes and miRNAs

**DOI:** 10.1038/srep21577

**Published:** 2016-02-10

**Authors:** Vibhav Gautam, Archita Singh, Sharmila Singh, Ananda K. Sarkar

**Affiliations:** 1 National Institute of Plant Genome Research (NIPGR), Aruna Asaf Ali Marg, New Delhi 110067, India.

## Abstract

Laser Capture Microdissection (LCM) is a powerful tool to isolate and study gene expression pattern of desired and less accessible cells or tissues from a heterogeneous population. Existing LCM-based methods fail to obtain high quality RNA including small RNAs from small microdissected plant tissue and therefore, are not suitable for miRNA expression studies. Here, we describe an efficient and cost-effective method to obtain both high quality RNA and miRNAs from LCM-derived embryonic root apical meristematic tissue, which is difficult to access. We have significantly modified and improved the tissue fixation, processing, sectioning and RNA isolation steps and minimized the use of kits. Isolated RNA was checked for quality with bioanalyzer and used for gene expression studies. We have confirmed the presence of 19-24 nucleotide long mature miRNAs using modified stem-loop RT-PCR. This modified LCM-based method is suitable for tissue specific expression analysis of both genes and small RNAs (miRNAs).

Isolation of high quality RNA is one of the most important prerequisite for analysis of a small tissue or cell population specific genes expression and their functional elucidation. The fluorescence-activated cell sorting (FACS) and laser capture microdissection (LCM) are the two recent powerful techniques that prevail the previously used manual microdissection method to study tissue specific gene expression^1^. In FACS, RNA is isolated from sorted cells, labelled with a fluorescence marker, such as Green Fluorescent Protein (GFP) and used for downstream application^2^,^3^,^4^,^5^. This highly efficient procedure, however, is limited by the availability of desired cell type specific molecular marker, anatomy and accessibility of the tissue, as well as by the vulnerability of isolated plant protoplasts to damage. To overcome these difficulties, LCM had been introduced to provide the flexibility to observe a specific population of cells under microscope, mark them on screen, microdissect and collect them in a collection tube or cap; RNA isolated from collected cells is used for downstream application^6^,^7^(Figs 1 and 2A,B). LCM-based approach was first used for functional genomics of cancerous tissues^6^,^8^. LCM coupled with next generation sequencing (NGS) or microarray and quantitative RT-PCR (qRT-PCR) are modern approaches for elucidating a cell or tissue specific global gene expression pattern^6^,^7^,^9^. LCM-based functional genomics (LCM-FG) approach has been used to study the comparative transcriptome of the shoot apical meristem (SAM), root apical meristem (RAM) and emerging leaf primordia in maize and *Arabidopsis*^9^,^10^,^11^,^12^. LCM-FG has also been used to discover the transcriptome specific to less accessible gametophytic cells of higher angiosperms or plant pathogen interaction^13^,^14^,^15^,^16^.

For LCM of soft and less accessible tissue (with cell walls in plants), it is necessary to fix, embed (in wax) and make thin sections. The common challenges faced during standard LCM-based method are the poor quality of RNA, low quantity and absence of 20–24 nt mature small RNAs (such as miRNAs) in the RNA sample, thereby reducing the efficacy and increasing the limitations of the concerned downstream experiments, such as NGS or microarray. Expensive kits commonly used to isolate RNA from paraplast embedded tissue, produce low yield, no or insufficient small RNAs and make the experiment less affordable. Although amplification of LCM tissue-derived RNA can increase the RNA amount, other problems still persist. The quality and quantity of LCM-derived RNA are mostly affected by the tissue fixation, tissue handling during sectioning as well as by post-LCM RNA isolation procedure. To overcome aforesaid difficulties, we have significantly improved and optimized the tissue fixation, sectioning, RNA isolation and amplification steps by modifying the existing protocols^7^,^9^,^17^,^18^. Our protocol was able to isolate high quality RNA from paraplast embedded tissue, as evident from good RNA integrity number (RIN, Fig. 2C). Using RT-PCR and modified stem-loop RT-PCR methods, we have shown the expression of selected genes and mature miRNAs in the embryonic RAM (Fig. 3A,B). Therefore, our protocol was able to isolate total RNA, which included mature miRNAs, from LCM-derived embryonic RAM of *Arabidopsis* (Fig. 3B). As we have minimized the use of kits, the total RNA thus obtained is suitable for more efficient and cost-effective LCM-FG studies. Schematic representation of the entire protocol is outlined (Fig. 1), which we have modified at various steps to improve the quality and quantity of RNA. To evaluate the efficiency of our method, we have compared three other existing protocols^7^,^9^,^17^,^18^ in parallel with ours, and observed that our modified and optimised protocol is better than the existing ones. Using our protocol we could isolate good quality of RNA including miRNA with higher yield. A comparative analysis of these four protocols is mentioned in Table 1.

We fixed dissected *Arabidopsis* silique tissue (harboring embryos inside ovules or seeds) using acetone (100%). For better penetration of the fixative inside the plant tissue and to minimize degradation of cellular RNA, soon after (within 15 minutes) harvesting (in acetone), tissues were put under vacuum infiltration for minimum 15 minutes or till they settle at the bottom of the tube indicating that fixative has completely entered the tissues and replaced internal air. We replaced the old fixative with fresh acetone once, incubated overnight at 4 °C, with continuous shaking. Next morning samples were passed through 3:1, 1:1, and 1:3 gradients of acetone: xylene for one hour each, followed by one change with 100% xylene. We have reduced the number and durations of these steps, instead of commonly practiced multiple changes with acetone and longer incubation^7^,^9^,^18^. To avoid the probable damage of RNA quality during prolonged incubation at high temperature, we incubated tissue samples for paraplast (wax) infiltration at a reduced temperature of 57 °C, which is just above the melting temperature of paraplast, instead of commonly practiced 60 °C^7^,^9^,^18^. Tissue blocks of appropriate size and orientation were sectioned using microtome to obtain strips of 8–10 μm thick sections, which were flattened by floating for 3–5 minutes on RNase free water at 50–55 °C. We took the flattened tissue sections on to RNase free charged slides and dried at 42 °C for maximum 30 minutes (instead of overnight^7^,^9^,^18^) to attach the tissue to the slide surface. We obtained high quality of RNA with good RIN value (Fig. 2C) using this and aforesaid optimized conditions. We have observed that common histological practices of tissue flattening at 40–42 °C and drying at 42 °C for prolonged period (an hour- overnight)^7^,^9^,^18^ deteriorate the quality of RNA as evident from low RIN value (Fig. 2C).

For LCM, tissue sections were dewaxed by dipping the slides in ‘histoclear’ solution and air dried. Embryonic RAM, the tissues of our interest, were marked on screen (Fig. 2A), microdissected out, and collected in the cap of 0.5 ml microfuge tubes using LCM system. We have isolated RNA from 50 sections (5 sections/embryo) of embryonic RAM (as marked, Fig. 2B). Common methods for RNA isolation from LCM-derived tissue (paraplast embedded) involve kits that produced low yield and no or insufficient small RNAs^7^,^9^,^17^,^18^. To reduce the RNA isolation cost while maintaining the quality and also to isolate ample amount of small RNAs, we optimized the RNA isolation method using TRIzol reagent (Thermo Fisher Scientific,USA) based procedure (methods). Our method of RNA isolation could overcome the aforesaid limitations and isolate high quality RNA including small RNAs (Figs 2C and 3B). We checked the quality of RNA isolated from three biological replicates of LCM-derived tissue using Bioanalyzer (Agilent). Our results showed that the RNA isolated from tissues processed through our optimized procedure was of high quality, as evident by high RIN value average ~8.1) (Fig. 2C, Table 1). On the other hand, RNA isolated from tissues processed through previously described common methods^7^,^9^,^17^,^18^ was of poorer quality, as evidenced by low RIN value (~6.3–~7.2).

For the downstream application that requires very high amount of RNA, the LCM-derived RNA can be amplified using any commercially available kit or with manual amplification method^18^,^19^. However, amplification process may leads to some degree of variation in the relative transcript abundance, due to the non-linear amplification of transcripts^18^,^19^. Since our improved protocol produces good yield of RNA, amplification of RNA should not be necessary for downstream processes with moderate range of RNA requirement. For our stem loop RT-PCR of miRNA, no amplification of RNA (obtained through our method) was necessary.

To investigate if the isolated RNA is good enough for gene expression studies, we synthesized cDNA from amplified LCM-derived RNA obtained from embryonic RAMs. Since several genes, phytohormones, epigenetic factors, and miRNAs have been implicated in plant root development, few candidate genes and miRNAs were selected for their expression in *Arabidopsis* RAM^20^,^21^,^22^. Expression of constitutively expressed genes such as *ACTIN4 (ACT4), TONOPLAST INTRINSIC PROTEIN (TIP41), ELONGATION FACTOR1α (EF1α)* and *At2g28390*, and tissue specific *WUSCHEL RELATED HOMEOBOX5 (WOX5)* and *SHOOT MERISTEMLESS (STM)* genes were studied using RT-PCR analysis. All these genes, except *STM*, were expressed in the embryonic root apical meristem, as indicated by single sharp band in gel electrophoresis (Fig. 3A). Expression of root quiescent center (QC) specific *WOX5*^23^,^24^ and absence of SAM specific *STM*^25^ in the embryonic RAM confirmed that the RNA isolated using our method was RAM specific and without contamination from distantly related tissue^24^,^26^. This suggests that the high quality RNA obtained through our method is suitable for tissue specific gene expression studies.

We further investigated if the LCM-derived RNA isolated through our optimized method also contained small RNAs, such as miRNAs, using stem-loop RT-PCR for mature miRNA. We used combination of stem-loop primers and miRNA specific primers (Table 2) for stem loop RT-PCR based expression analysis (Experimental procedures, Fig. 4) of miRNAs such as *miR164*, *miR165*, *miR390, miR842*, and *miR172*, which were previously reported to be expressed in root^21^,^27^,^28^. As expected, the bands of approximately 50–60 bp long amplicon of miRNAs were obtained (Fig. 3B). Our results showed that all these miRNAs, which were previously reported in post-embryonic root^21^,^27^,^28^, are also expressed in embryonic RAM. This indicates the potential role of these miRNAs in the establishment or maintenance of embryonic RAM in embryo development. We have also confirmed the suitability of LCM-derived RNA for tissue specific miRNA expression profiling using RT-PCR, stem loop RT-PCR and microarray analysis (data not shown). Thus, our results confirm that LCM-derived RNA, isolated through our improved method also contains mature miRNAs and therefore, is suitable for studying tissue specific expression of miRNAs.

With the goal to improve the quality and yield of LCM derived RNA, to isolate small RNA, and to make the procedure cost-effective, we have developed a new protocol by modifying and optimizing mainly three existing methods^7^,^9^,^17^,^18^. To validate the efficiency of our method, we have made a comparative study using these four protocols (including ours) for LCM-based RNA isolation, and assessment of quality and quantity of RNA (Fig. 2C; Table 1). The major modification steps involved tissue fixation, tissue processing for dehydration/infiltration, preparation of slides and sections, and RNA isolation (Fig. 1; Table 1). For tissue fixation, we have used acetone, instead of ethanol-acetic acid (E:AA) or formaldehyde-acetic acid-ethanol (FAA)^7^,^17^. Since long dehydration/infiltration steps, as used in several protocols^7^,^9^,^18^, may subject the tissue vulnerable to RNA degradation, we have minimized the total dehydration/infiltration time to 4 hours (see Table 1 to compare with others’). To maintain good integrity of tissue and RNA, which are more prone to damage at high temperature, we have performed paraplast exchanges at a reduced temperature of 57 °C, instead of commonly used temperature of 58–60 °C^7^,^9^. It has been reported that the incubation of slides and tissue sections for long duration at high temperature reduces the RNA quality^17^. Therefore, we have avoided overnight/long hours of drying of slides/tissue sections, as followed in a few common methods^7^,^9^,^18^. Instead, in our protocol, tissue sections were collected on RNase free charged slides and flattened at the 50–55 °C for 3–5 minutes and followed by dried at 42 °C for 30 minutes only. Above mentioned commonly used protocols used expensive column-based kits such as ‘Pico Pure kit’, for isolation of RNA from LCM-derived tissues^7^,^9^,^17^,^18^. In our hands, these protocols failed to obtain any miRNA fraction (neither there is any report for that) (Fig. 3B), which probably was because this kit was not designed to retain small sized RNAs and also due to the poor RNA yield. In our protocol, we have used optimized manual method of RNA extraction (see below), which was capable enough to isolate the miRNAs from the LCM derived tissues (Fig. 3B). Besides this, our protocol, which used TRIzol based method of RNA extraction, produced RNA of better yield and quality, as compared to other protocols (Table 1). Moreover, since we have minimized the use of kits, our protocols is more cost effective.

To our knowledge, this is the first report of tissue specific miRNA isolation and expression analysis using LCM-based method in plants. Although, here we have described the method for plants, it may be useful for animal studies as well. Thus, our optimized LCM-based method should be more useful as it is cost-effective and suitable for broader applications including small RNA studies in plants. Since, miRNAs are relatively conserved among various plants and diverged only infrequently^29^,^30^, our improved method could be very efficient and useful for studying tissue specific miRNA expression profiling in many model and non-model plants.

## Experimental Procedures

RNase free condition (tools, containers, solutions and handling) should be used in all the following steps of the method from tissue collection, fixation, embedding, LCM through RNA isolation and downstream experiments.

### Tissue fixation and processing

*Arabidopsis* siliques containing embryos were dissected out using the RNase free scalpel, trimmed at the ends and were immediately immersed into the ice cold 100% acetone (Qualigen, USA), a fixative, in 2 ml RNase free microfuge tubes and were put under vacuum. For better penetration of the fixative, vacuum infiltration was done at 4 °C under 350 mm Hg pressure for 15 mins or until all the siliques settled completely at the bottom of the tube. After vacuum infiltration the old fixative was replaced by the fresh ice cold acetone (100%) and kept at room temperature (RT) with mild agitation for 1 hr. A 2^nd^ replacement of fixative was done with fresh ice cold 100% acetone and left overnight at 4 °C with gentle agitation. Due to histological differences and variation in secondary metabolites among different plant tissues, fixation steps may need to be standardized. Next day, tissues were dehydrated/infiltrated by passing through the series of acetone: xylene (Qualigen, USA) of 3:1, 1:1, 1:3 ratios for 1 hr each and was followed by replacement of solution with pure xylene (100%) and incubated for 1 hr at RT with agitation. Few paraplast chips (Sigma Aldrich, USA) were added into the vial and left overnight at RT. Next day the tubes were kept at 57 °C oven to melt the paraplast; old paraplast was exchanged with the fresh molten paraplast at 57 °C twice in a day, for the period of three days.

### Tissue embedding in paraplast

After six changes of paraplast at 57 °C, siliques were embedded using the Peel A-Way moulds (Sigma Aldrich, USA) or steel mould (Yorco, India). Contents of a vial were dispensed in a mould placed on a hot plate at 57 °C and siliques were arranged in a desired orientation making small blocks using RNase free forceps. The paraplast was solidified by placing the moulds at room temperature (RT) and stored at 4 °C.

### Tissue sectioning

Tissue blocks were trimmed into a desired shape and placed in desired orientation on the plastic embedding rings (Himedia, India). Rings were fixed with the holding clamp of the rotary microtome (Leica RM2265) and 8–10 μm thin tissue sections were made. Tissue sections were flattened for 3–5 mins on water at 50–55 ^o^ C (below the melting temperature of paraplast used), taken on HistoBond + charged slides (Marienfeld, Germany), dried for maximum 30 mins on hot plate at 42 °C, and stored temporarily at 4 °C until LCM was performed. Since the incubation temperature of tissues may affect RNA quality^17^, we have modified the temperature accordingly, as mentioned above.

### Laser Capture Microdissection (LCM)

Tissue sections were dewaxed by dipping the slides twice in histoclear (Histochoice clearing reagent, Sigma Aldrich, USA) for 2 mins and air dried at RT. Initial observation of the tissues were done under stereo microscope. Slides were observed under LCM microscope, embryos were identified, RAMs were marked using on screen tool of PALM MicroBeam (Carl Zeiss, Germany). Tissues were laser cut along the marking, and followed by catapulting. LCM-based catapulted tissues were collected in RNase free 0.5 ml tubes having a drop of mineral oil (Amresco,USA) and stored temporarily at –80 °C or directly used for RNA isolation.

### RNA isolation

Collection tubes containing LCM-derived tissues were centrifuged at 5000 rpm for 1 min, 150 μl of TRI-reagent (Sigma Aldrich, USA) was added and centrifuged at 5000 rpm for another 2 mins. 100 μl of chloroform (Qualigen, USA) was added to the tube and followed by brief vortexing, incubation at RT for 15 minutes, and centrifugation at 13000 rpm for 30 mins. The upper aqueous phase was taken into a fresh 1.5 ml microfuge tube (Axygen, India); equal volume of isopropanol (Qualigen,USA) was added, mixed and kept at −20 °C for 1 hr, and followed by centrifugation at 13,000 rpm for 1 hr. The supernatant was discarded and the pellet was washed with 100 μl of 70% ethanol by centrifuging at 7500 rpm for 15 mins. The pellet was air dried and finally dissolved in 10 μl of nuclease free water (Sigma). The concentration and RIN of the RNA samples were checked using Nanodrop 1000 (Thermo Fischer Scientific, USA) and Bioanalyzer nanochip (Agilent, 2100), respectively.

### RT-PCR and stem-loop RT-PCR

Approximately, 1 μg of RNA was treated with DNase I (Fermentas, USA) as per company’s manual, heat inactivated at 65 °C for 10 mins. DNase I treated RNA was used to make 1^st^ strand cDNA using SuperScript III (Thermo Fischer Scientific, USA; Originally Invitrogen) as per company manual; 2 μl of diluted (5×) cDNA was used for each 20 μl of PCR reaction using gene specific primers. The PCR program used: 94 °C for 5 mins:1 x; 94 °C for 15 secs, 60 °C for 40 secs:40×; 72 °C for 1 min, 72 °C for 10 mins and holding at 4 °C (Veriti, Applied Biosystems, USA). PCR products were checked by gel electrophoresis.

**Figure 3:**
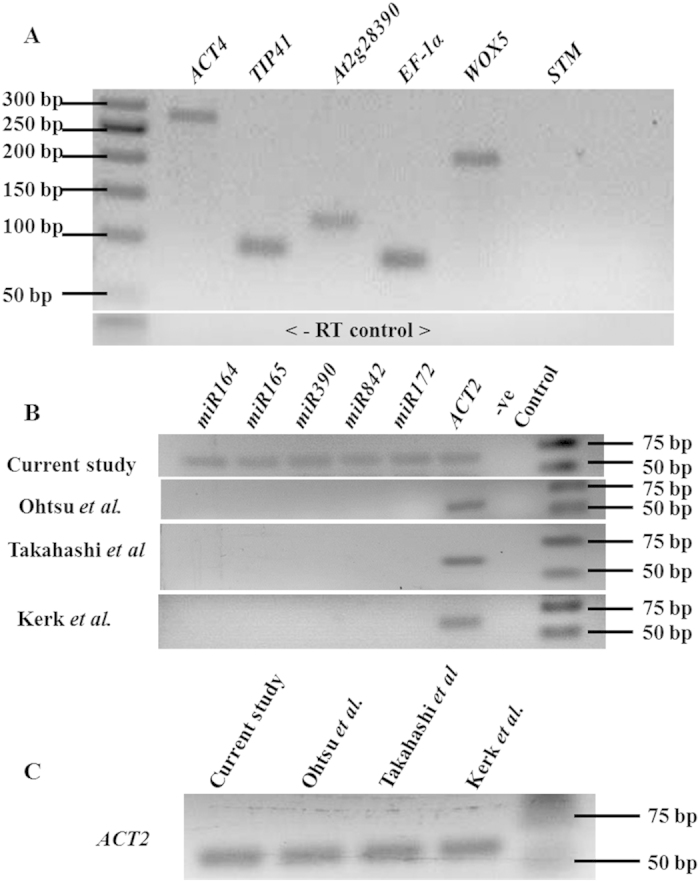
Expression analysis of selected genes and miRNAs using RT-PCR and stem loop RT-PCR, respectively. **(A)** RT-PCR showing the expression of constitutive *ACT4, TIP41, At2g28390, EF-1α*, and root specific *WOX5* genes; no expression of SAM specific *STM* was observed in RAM derived RNA indicating no tissue contamination. Lower panels: minus RT control. **(B)** Stem-loop RT-PCR, showing the expression of mature *miR164*, *miR165*, *miR390, miR842*, *miR172*, and *ACT2*. **(C)** *ACT2* normalization for semiquantitative RT-PCR.

**Figure 4:**
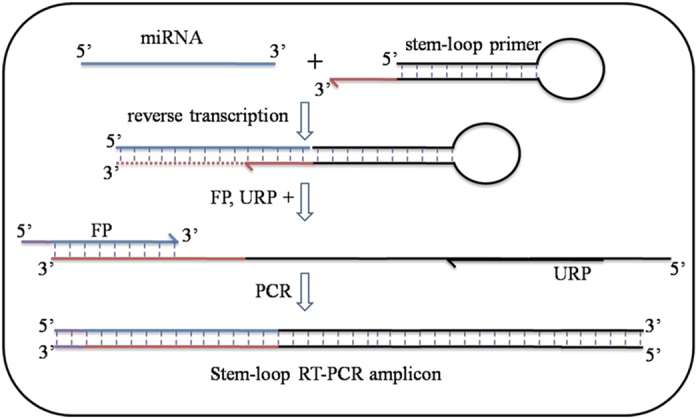
Illustration of stem-loop RT-PCR method to amplify mature miRNAs. At first, mature miRNAs are reverse transcribed using stem-loop primer (SLP). Forward primer (FP; miRNA specific, with 5′ overhang) and universal reverse primer (URP)^31^ are used to PCR-amplify the RT-product either through end-point PCR or quantitative PCR.

Stem-loop RT-PCR was used to detect and amplify mature miRNAs. Stem-loop reverse primer (SLP) was designed such that it formed a hair-pin and possessed a 3′ overhang complementary to the miRNA (Fig. 4). A miRNA specific forward primer (FP) with 5′ adapter (to match temperature with other primer) and a universal reverse primer (URP) were designed for PCR amplification (Fig. 4)^31^,^32^. For reverse transcription, 11.5 μl ‘Reaction mix-A’ containing 0.5 μl of 10 mM dNTPs, DNase I treated RNA (100 ng) and water was heated at 65 °C for 5 mins, cooled on ice for 2 mins, and added into 6.5 μl of ‘reaction mix-B’ containing first strand buffer (1×), 2 μl of 0.1 M DTT, 10 U of RNase OUT and 50 U of SuperScript III (Invitrogen); 1 μl each of 1 μM SLP and 1 μM control reverse primers were added. Reaction was performed in a Thermal Cycler (Veriti, Applied Biosystems) with program: 16 °C for 30 mins ×1; 30 °C for 10 secs, 42 °C for 10 secs, 50 °C for 1 sec: 60 x; 85 °C for 5 mins: 1× followed by incubation at 4 °C^31^,^32^. RT-PCR based expression of analysis of miRNAs was done using standard end-point PCR (program: 94 °C for 5 mins: 1 x; 94 °C for 30 secs, 60 °C for 40 secs: 35 x; 72 °C for 1 min, 72 °C for 10 mins and holding at 4 °C) (Figs 3 and 4) or quantitative RT PCR (as above, data not shown). The 20 μl of an end-point PCR reaction includes miRNA specific FP (0.25 μM), URP (0.25 μM), dNTPs (0.2 mM), 3B DNA Polymerase (0.5 U) (Black Biotech, India), buffer, water and 1 μl of stem-loop RT product. PCR products were checked by agarose (3%) gel electrophoresis.

## Additional Information

**How to cite this article**: Gautam, V. *et al.* An Efficient LCM-Based Method for Tissue Specific Expression Analysis of Genes and miRNAs. *Sci. Rep.* **6**, 21577; doi: 10.1038/srep21577 (2016).

## Figures and Tables

**Figure 1:**
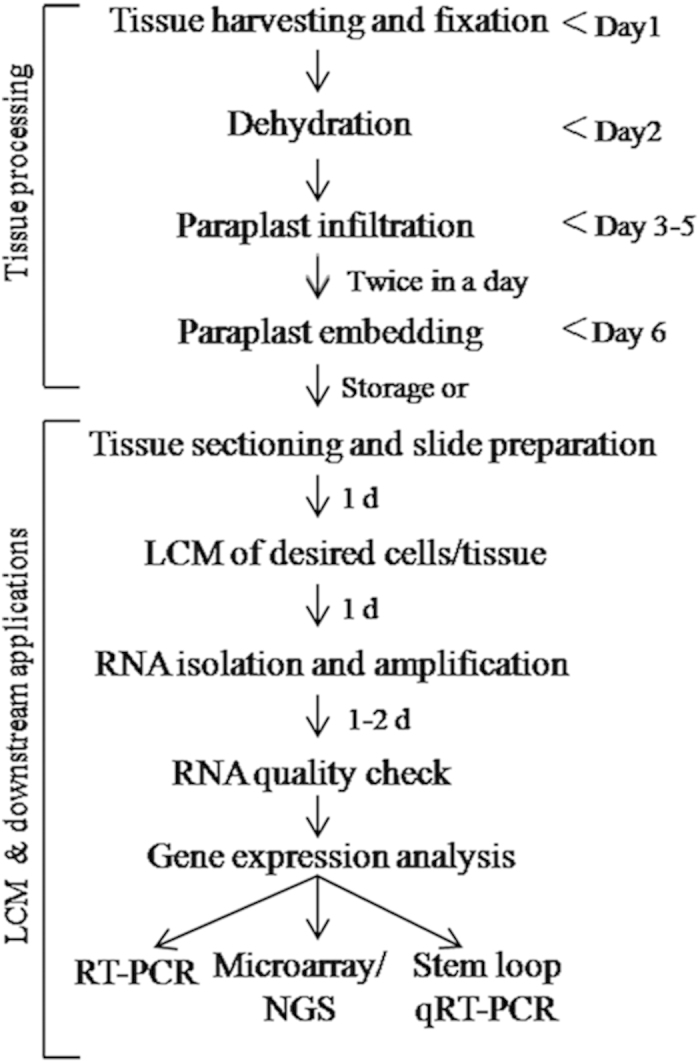
Schematic flowchart of the LCM based tissue specific RNA isolation and gene expression study. Flowchart is divided into two parts: tissue processing, LCM and its downstream applications. Flowchart indicates the predicted timing involved at each step of the experiment. ‘d’ indicates day.

**Figure 2:**
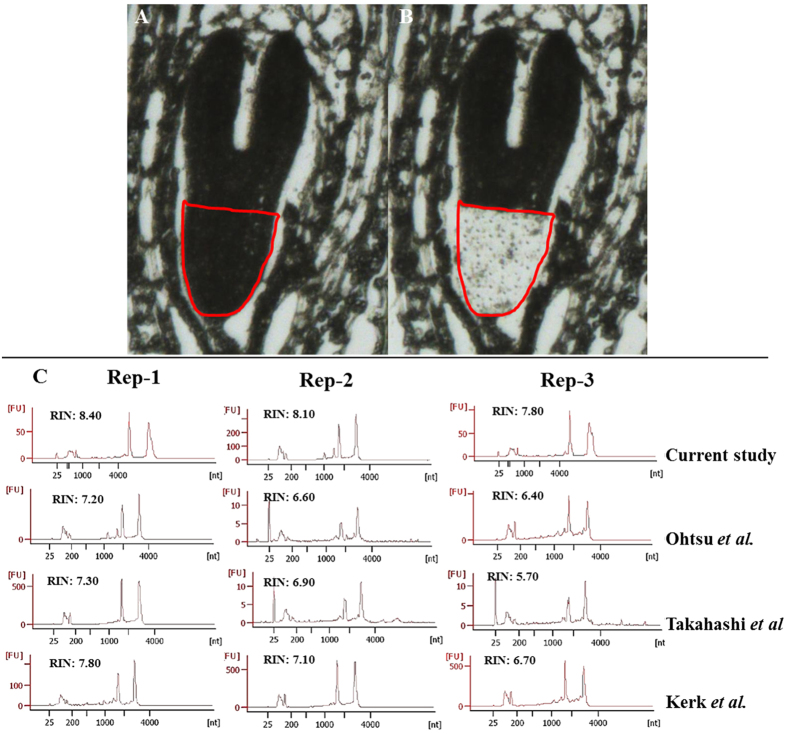
RNA isolation from LCM-derived tissue and the quality check using bio analyzer. **(A)** Embryonic RAM of *Arabidopsis thaliana* before (marked with red outline) and, **(B)** after LCM **(C)** Bioanalyzer-based analysis of LCM-tissue derived RNA. (Rep indicates replicate) using four different methods. RIN value shown for the result of each replicate indicates RNA quality.

**Table 1:** Comparison between various protocols for tissue fixation, LCM and RNA isolation.

Description of the protocols	Kerk *et al.*^7^	Takahashi *et al.*^17^	Ohtsu *et al*^9^*&*Scanlon *et al.*^18^	Current study
Fixative (at 4^o^C)	3:1 Ethanol : Acetic acid	75% Ethanol: 25% Acetic acid	100% Acetone	100% Acetone
Dehydration /Infiltration	Dehydration in 75%, 85%, 100%,100%,100% ethanol for 3 hrs at RT. Infiltration in ethanol: xylene 75%:25%,50%:50%,25%:75%,100%,100%,100% for 3 hrs, each at RT	Dehydration in 70%, 80%, 90%, 100% and absolute ethanol at 58^o^ C for 1 min 30 secs. Infiltration in 50% ethanol/ 50% n-butanol, 50% acetone/ 50% n-butanol, 50% acetone/ 50% n-butanol, 100 % n-butanol at 58 ^o^ C for 1 min 30 secs each.	Dehydration in 3:1, 1:1, and 1:3 in acetone: xylene at RT for 1.5 hrs each followed by three changes with 100% xylene at RT for 1 hr.	Dehydration in 3:1, 1:1, and 1:3 in acetone: xylene followed by 100% xylene at RT for 1 hr.
Paraplast exchange temperature	58^o^ C for 6 hrs, 2 days	58 °C for 30 mins, 4 times	60 °C for 3 times in a day, 2 days	57 °C twice in a day, 3 days
Temperature and duration for tissue flattening	42 °C, till fully stretched.	57 °C for 5 mins.	40 °C for 5–20 mins.	50–55 °C for 3–5 mins.
Temperature and duration for slide drying	Air dried for overnight.	Dried at 42 °C for 20 mins.	Dried at 42 °C for overnight.	Dried at 42 °C for 30 mins.
Method of RNA isolation	Pico Pure kit/ Nanoprep kit/ kit/TRIzol	Pico Pure kit	Pico Pure kit	TRIzol- based method (no kit)
RNA yield (same no. of tissue sections & elution volume in each)	25-30 ng/μl (using Pico Pure Kit )	23–30 ng/μl	25–35 ng/μl	100–160 ng/μl
miRNA expression	Absent	Absent	Absent	miRNA present
Avg. RIN value (03 replicates)	7.20	6.30	6.70	8.10

**Table 2:** List of primers used in the study.

Gene Name	Primer Sequences
*ACTIN4*-F	GTATGTTGCCATTCAAGCTGTTC
*ACTIN4*-R	GCGTAACCCTCGTAGATTGGTA
*TIP41*-F	GGGTATCCAGTTGACTTAGCAG
*TIP41*-R	GGGATCTTCAGTTTCTGTGTCG
*AT2G28390-F*	TTCTATGTTGGGTCACACCAG
*AT2G28390-R*	CACTTCATTCTCCACATCTTTTACC
*EF-1α*-F	GATTGCCACACCTCTCACATTGCAG
*EF-1α*-R	GCTCCTTCTCAATCTCCTTACCAG
*WOX5*-F	GATTGTCAAGAGGAAGAGAAGGTGA
*WOX5*- R	AGCTTAATCGAAGATCTAATGGCG
*STM*-F	GAAGCTTACTGTGAAATGCTCG
*STM*- R	AACCACTGTACTTGCGCAAGAG
miR164-FP	GCGGCGGTGGAGAAGCAGGGCA
miR164-SLP	GTTGGCTCTGGTGCAGGGTCCGAGGTATTCGCACCAGAGCCAACTGCACG
miR165-FP	CGGCGGTCGGACCAGGCTTCA
miR165-SLP	GTTGGCTCTGGTGCAGGGTCCGAGGTATTCGCACCAGAGCCAACGGGGG
miR390-FP	GCGGCGGAAGCTCAGGAGGGAT
miR390-SLP	GTTGGCTCTGGTGCAGGGTCCGAGGTATTCGCACCAGAGCCAACGGCGCT
miR842-FP	GCGGCGGTCATGGTCAGATCCG
miR842-SLP	GTTGGCTCTGGTGCAGGGTCCGAGGTATTCGCACCAGAGCCAACGGATGA
miR172-FP	GCGGCGGAGAAUTCTTGATGATG
miR172-SLP	GTTGGCTCTGGTGCAGGGTCCGAGGTATTCGCACCAGAGCCAACATGCAG
URP	GTGCAGGGTCCGAGGT
*ACTIN2*-F	TCAGATGCCCAGAAGTCTTG
*ACTIN2*-R	GTGGATTCCAGCAGCTTCCA
